# Molecular phylogeny of one extinct and two critically endangered Central Asian sturgeon species (genus *Pseudoscaphirhynchus*) based on their mitochondrial genomes

**DOI:** 10.1038/s41598-020-57581-y

**Published:** 2020-01-20

**Authors:** Artem V. Nedoluzhko, Fedor S. Sharko, Svetlana V. Tsygankova, Eugenia S. Boulygina, Anna E. Barmintseva, Anna A. Krasivskaya, Amina S. Ibragimova, Natalia M. Gruzdeva, Sergey M. Rastorguev, Nikolai S. Mugue

**Affiliations:** 1 Nord University, Faculty of Biosciences and Aquaculture, Bodø, 8049 Norway; 2 National Research Center “Kurchatov Institute”, Moscow, 123182 Russia; 3 Institute of Bioengineering, Research Center of Biotechnology of the Russian Academy of Sciences, Moscow, 117312 Russia; 4 Russian Federal Research Institute of Fisheries and Oceanography, Moscow, 107140 Russia; 5 Koltzov Institute for Developmental Biology of the Russian Academy of Sciences, Moscow, 117808 Russia

**Keywords:** Molecular evolution, Phylogenetics, Evolutionary biology

## Abstract

The enigmatic and poorly studied sturgeon genus *Pseudoscaphirhynchus* (Scaphirhynchinae: Acipenseridae) comprises three species: the Amu Darya shovelnose sturgeon (*Pseudoscaphirhynchus kaufmanni* (Bogdanow)), dwarf Amu Darya shovelnose sturgeon *P*. *hermanni* (Kessler), and Syr Darya shovelnose sturgeon (*P*. *fedtschenkoi* (Bogdanow). Two species – *P*. *hermanni* and *P*. *kaufmanni* – are critically endangered due to the Aral Sea area ecological disaster, caused by massive water use for irrigation to support cotton agriculture, subsequent pesticide pollution and habitat degradation. For another species – *P*. *fedtschenkoi* – no sightings have been reported since 1960-s and it is believed to be extinct, both in nature and in captivity. In this study, complete mitochondrial (mt) genomes of these three species of *Pseudoscaphirhynchus* were characterized using Illumina and Sanger sequencing platforms. Phylogenetic analyses showed the significant divergence between Amu Darya and Syr Darya freshwater sturgeons and supported the monophyletic origin of the *Pseudoscaphirhynchus* species. We confirmed that two sympatric Amu Darya species *P*. *kaufmanni* and *P*. *hermanni* form a single genetic cluster, which may require further morphological and genetic study to assess possible hybridization, intraspecific variation and taxonomic status and to develop conservation measures to protect these unique fishes.

## Introduction

The animal diversity and classification remain complex with new species being continuously discovered and described. Meanwhile, an increase in human activity has led to the degradation of ecosystems, the destruction of native habitats and the direct extinction of many animal species^1^.

Museum specimens have become increasingly more valuable in evolutionary and conservation biology studies solving fundamental and applied problems using a novel arsenal of molecular genetic techniques. DNA isolation from historical samples and high-capacity DNA sequencing open up new opportunities associated with the investigation of evolution and phylogeny of extinct or endangered species^2–6^.

The order Acipenseriformes is an ancient fish group which is represented by two families: Polyodontidae and Acipenseridae^7^. All of the extant species from the Acipenseridae family that inhabit North America and Eurasia are endangered and listed in CITES appendices^8,9^.

The Aral Sea basin in Central Asia (Kazakhstan, Tajikistan, Turkmenistan and Uzbekistan) has historically had an extraordinary endemic fauna, including four acipenserid species: the Amu Darya shovelnose sturgeon (*Pseudoscaphirhynchus kaufmanni* Bogdanov, 1874), dwarf Amu Darya shovelnose sturgeon (*P*. *hermanni* Kessler, 1877), Syr Darya shovelnose sturgeon (*P*. *fedtschenkoi* Kessler, 1872), and Aral ship sturgeon (*Acipenser nudiventris* Lovetsky, 1828), with the two latter species possibly extinct in their native habitat, and, unlike *A*. *nudiventris*, *P*. *fedtschenkoi* also does not exist anywhere in aquaculture^10–12^. The species’ extinction in this region over the recent decades is mostly associated with the Aral Sea ecological disaster that was initially caused by anthropogenic activity through intensive water use from the Amu Darya and the Syr Darya rivers for irrigation and cotton agriculture development as well as wide application of pesticides and mineral fertilizers^13^.

**Figure 1:**
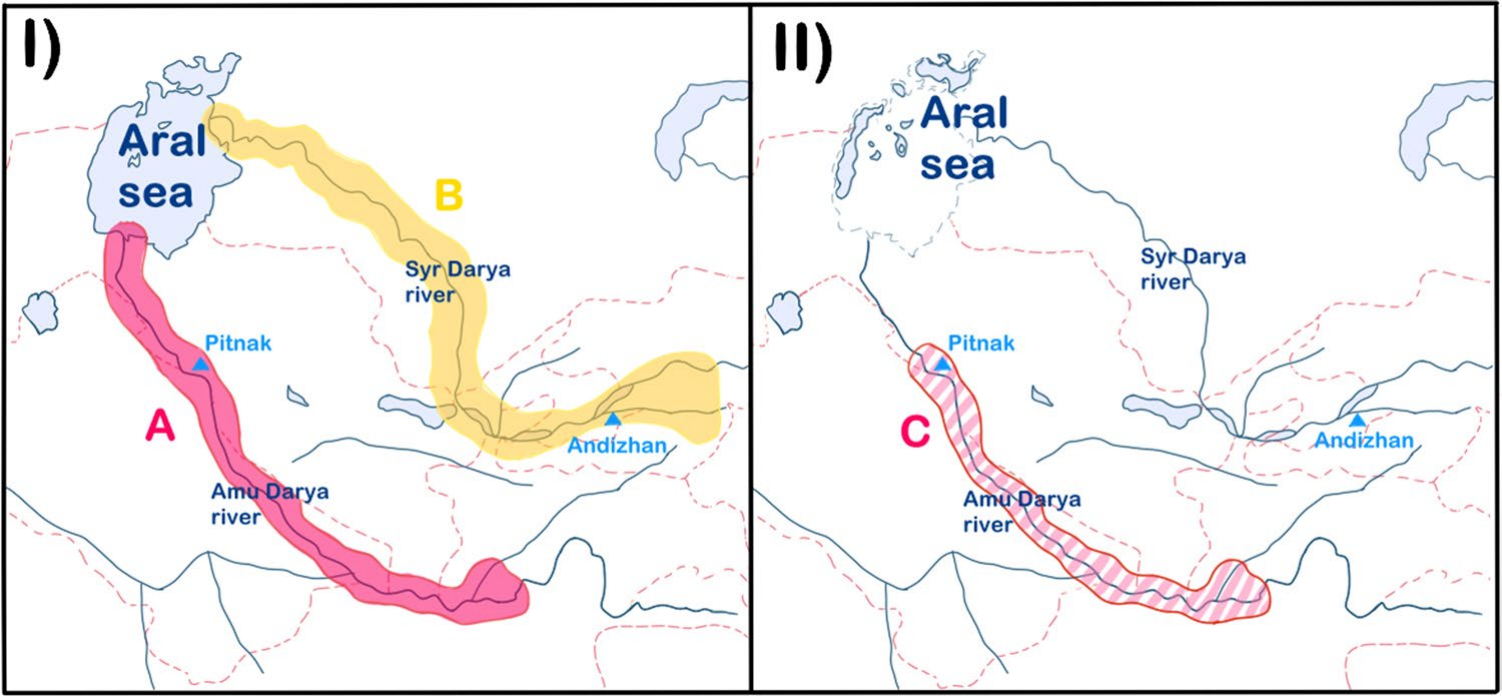
Distribution map of *Pseudoscaphirhynchus* species investigated in our study (I – 1960s years; II – 2010s years). A – Former distribution of Amu Darya shovelnose sturgeons *P*. *kaufmanni* and *P*. *hermanni;* B – Former distribution of the extinct Syr Darya shovelnose sturgeon *P*. *fedtschenkoi*. C – Presumable current distribution of Amu Darya shovelnose sturgeons *P*. *kaufmanni* and *P*. *hermanni*.

The *Pseudoscaphirhynchus* genus (Scaphirhynchinae: Acipenseridae) is represented by three freshwater sturgeon species that have limited distribution in Central Asia. One of them – Syr Darya shovelnose sturgeon (*P*. *fedtschenkoi*) – has not been observed since the 1960s, while the other two – *P*. *kaufmanni* and *P*. *hermanni* – are endemic and critically endangered species that live in the muddy waters of the Amu Darya and Panj river basins and are poorly studied because of their rarity^14,15^ (Fig. 1).

Traditionally, Central Asian shovelnoses were placed in the Scaphirhynchinae subfamily together with three *Pseudoscaphirhynchus* species from North America. Based on recent morphology and molecular genetics studies, Scaphirhynchinae were reclassified as paraphyletic taxa, and Asian species were transferred to Asipenserinae subfamily, placing these species as a sister group to the stellate sturgeon (*Acipenser stellatus*)^14–16^.

Previously, the information on the phylogenetic status of *P*. *hermanni* and *P*. *kaufmanni* was based on morphological traits^17^ and on the sequences of the mitochondrial *cytB* gene, showing a monophyletic origin for both of them^10^. At the same time, molecular phylogenetic status of extinct *P*. *fedtschenkoi* remained unclear because it had not been caught for more than fifty years in the Syr Darya basin and can only be found in few museum collections^12^.

In this study, we present a comparative analysis of four mitochondrial genomes, which were assembled from DNA extracted from museum specimen tissue of extinct *P*. *fedtschenkoi* and ethanol-fixed tissues of extant *P*. *kaufmanni* and *P*. *hermanni*. Our results show that the Central Asian shovelnose species form a monophyletic group and Amu Darya sturgeons demonstrate a significant divergence from the locally extinct Syr Darya sturgeon.

## Results and Discussion

We obtained four complete mitochondrial genomes of shovelnose sturgeon specimens, belonging to three *Pseudoscaphirhynchus* species. The Next Generation sequencing (NGS) statistics on generated and mapped Illumina reads is presented in Table 1. For all NGS-studied specimens, including the extinct Syr Darya shovelnose sturgeon, the average sequence coverage is high (from 7028X to 10859X). The read length distribution for NGS-libraries (including the museum sample) is presented in Supplementary Figs. S1–S3. Assembled mitogenomes of *Pseudoscaphirhynchus* species were deposited to the GenBank (BioProject PRJNA472690).

**Table 1:** Illumina generated reads and the mitochondrial genome size of the *Pseudoscaphirhynchus* species.

Species name	Abbreviation	No. Illumina reads generated	Mitogenome size, bp	NCBI number
*P. fedtschenkoi*	FED01	116,758,094	16,613	SAMN09240668
*P. kaufmanni*	KAU03	161,089,296	16,615	SAMN09240696
*P. kaufmanni*	KAU02	Sanger sequencing	15,715	SAMN09829629
*P. hermanni*	HER01	180,707,170 (Sanger sequencing was also conducted)	16,640	SAMN09240697

Assembled mitogenomes of *P*. *fedtschenkoi*, *P*. *kaufmanni* and *P*. *hermanni* consist of 16,613 bp, 16,615 bp and 16,640 bp, respectively (Table 1), and contain 13 protein coding genes (PCGs), 2 rRNA genes, and 22 tRNA genes as was shown previously for other sturgeon mitogenomes^18^.

**Figure 2:**
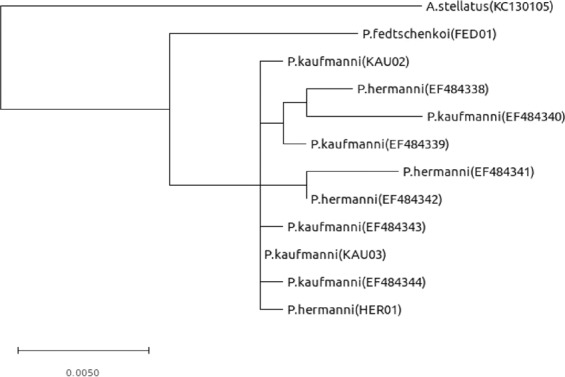
Maximum likelihood phylogenetic tree reconstruction of the *Pseudoscaphirhynchus* species, including an extinct Syr Darya shovelnose sturgeon based on nucleotide variability of *cytB* gene.

To evaluate genetic diversity among species of the *Pseudoscaphirhynchus* genus, we conducted phylogenetic analyses of the mitochondrial cytochrome B *(cytB)* gene using our project specimens and the ones published in the previous Amu Darya sturgeon study^10^. The maximum likelihood (ML) tree is shown in Fig. 2. Unlike Amu Darya species (*P*. *hermanni*, *P*. *kaufmanni*), located in the same cluster, Syr Darya *P*. *fedtschenkoi* species is clustered apart from them.

**Figure 3:**
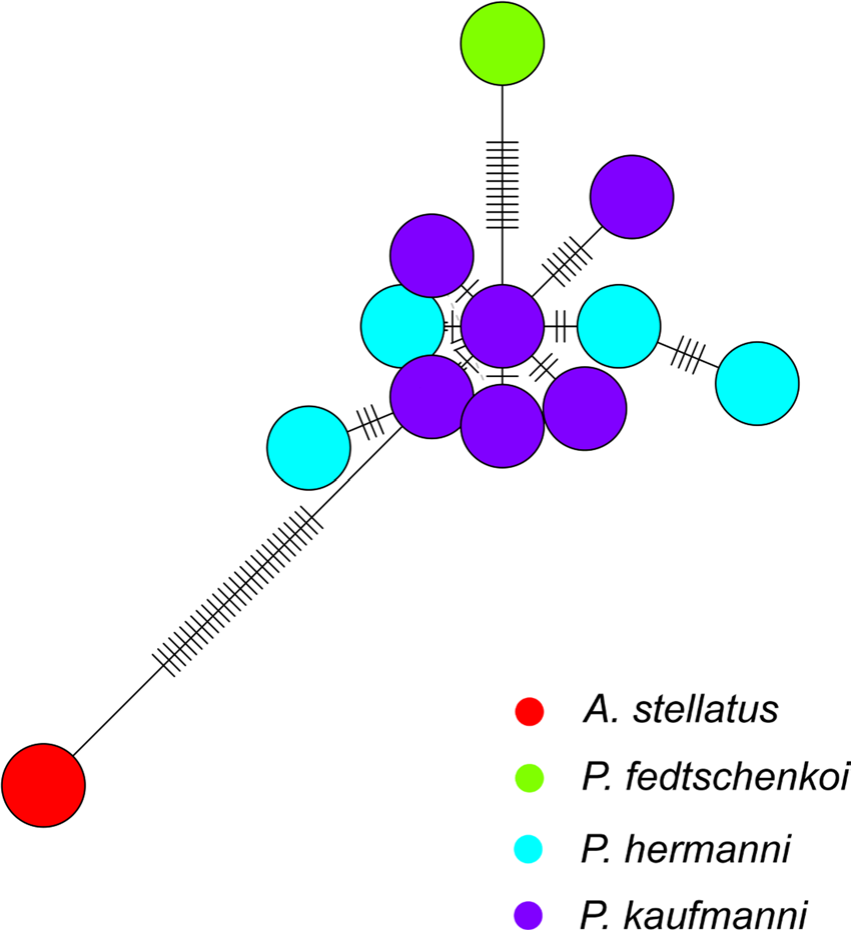
Mitochondrial DNA *cytochrome B* haplotype network of *Pseudoscaphirhynchus* species with *Acipenser stellatus* as an outgroup. Dashed lines indicate a number of distinguishing substitutions between the haplotypes.

The mixed clustering of the two Amu Darya species corresponds to the data from^10^ and can be explained as either incomplete lineage sorting (ILS) or recent hybridization. The haplotype network on Fig. 3 also suggests a mixed nature of Amu Daria *Pseudoscaphirhynchus* mitochondrial haplotypes and a larger genetic distance for the Syr Daria *P*. *fedtschenkoi* specimen.

Dillman *et al*.^10^ found that based on *cytB* gene analysis, Amu-Darya shovelnose and dwarf shovelnose sturgeons do not form distinct clusters, but the possible explanation proposed by the authors was the low rate of mitochondrial evolution. They suggested that complete genome sequencing of the sturgeons should be conducted in order to resolve this issue. Our complete mtDNA data also supports close similarity between two sympatric Amu Darya species, showing that samples KAU02 and HER01 are different by only 45 substitutions (0.27%). When the *cytB* region of these samples was extracted and compared with the sequences published by Dillman *et al*.^10^, it also supported lack of segregation between these two sympatric species.

**Figure 4:**
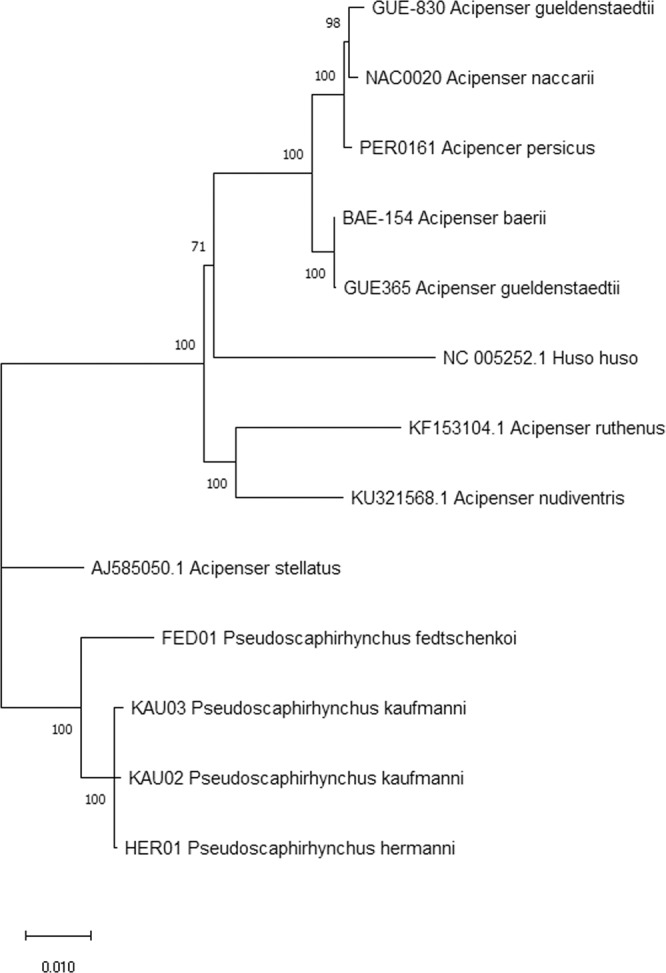
Maximum likelihood phylogenetic tree reconstruction of the Ponto Caspian sturgeon species, including the Amu Darya shovelnose sturgeon, dwarf Amu Darya shovelnose sturgeon, and extinct Syr Darya shovelnose sturgeon, based on their mitochondrial coding sequences (excluding *NADH4L* gene).

The mitogenomic phylogeny of *Pseudoscaphirhynchus* and other Ponto Caspian sturgeons was reconstructed based on our and previously published mitochondrial datasets. The control region as well as the *NADH4L* gene were discarded from the alignments as they produced phylogenetic noise and made the phylogenetic tree unstable (Fig. 4).

Mitochondrial DNA sequence analysis of the third species of this genus indicates that *P*. *fedtschenkoi* from the Syr Daria basin is clearly distinct from both Amu Darya species. Therefore, there is no support for previously suggested hypothesis of the slow evolutionary rate for mtDNA^10^. This finding raises the question about the taxonomic status of two Amu Darya species - *P*. *hermanni*, *P*. *kaufmanni*. The admixture of haplotypes can be explained by incomplete lineage sorting; although it is unlikely as the third species forms a distinct clade with high phylogenetic support. Another explanation could be a possible hybridization or mtDNA substitution, which would explain the absence of mtDNA lineage specific to *P*. *hermanni*. There is a third plausible explanation: the two Amu Darya species of shovelnose sturgeons, even though being morphologically very distinct^17,19^ (also see Table 2), are phenotypic morphs within a single species. Both of these currently recognized species are now present at the same time of year in the same part of Amu Darya river near Bukhara City (Uzbekistan) (Alexey Chernyak, pers. comm), and are also sympatric throughout the Amu Darya river basin. Large morphological variations were described for *P*. *kaufmanni* in the past, including large and dwarf morphs^19^. Two other forms - proposed as intermediate forms of *P*. *kaufmanni* and *P*. *hermanni* - were reported by Nikolsky (1938, cited in Berg 1948)^17^.

**Table 2:** Morphological variation (min-max (average)) of three Central Asia shovelnose sturgeon species (after Berg 1948) and one museum specimen (Andizhan, Fergana valley of the Syr Darya basin) which we have identified as a *P*. *fedtschenkoi*.

	*P*. *kaufmanni* (after Berg 1968)	*P*. *hermanni* (after Berg 1968)	*P*. *fedtschenkoi* (after Berg 1968)	Specimen at the Andizhan museum (FED 01)
Dorsal scutes	10–14 (12)	9–13 (10–11)	15–22	19
Lateral scutes	30–38 (34.5)	31–39 (35)	37–46	36 visible plus 3–5 (?) on the caudal peduncle which is not present on the museum specimen)
Abdominal scutes	6–19 (7–8)	6–9 (7)	?	8
Rostrum spines	present	absent	absent	absent
Caudal filament	present	absent	absent or present	lost or absent
Skin fold at the anterial edge of pectoral fin	absent	present	present	present

Striking morphological variations are also observed in *P*. *fedtschenkoi*, where Berg described three morphs: 1) morpha *typical* with long rostrum and absent (or almost absent) caudal filament, 2) morpha *brevirostrum* with short rostrum and long caudal filament, and 3) morpha *intermedia* with longer rostrum and well-developed filament. According to Leo Berg, all three morphs are often found by fishermen in one catch^17^. These morphological variations are almost as drastic as the ones between dwarf and Amu Darya sturgeons, which makes it plausible to suggest that the latter two forms may belong to one polymorphic species. To test this hypothesis, microsatellite or genotype-by-sequencing analysis should be performed, to clarify the taxonomic status of these imperiled fishes.

The results of our study demonstrate that extinct species can be assessed using DNA sequencing of archived and museum samples and subsequent DNA sequence analysis making possible phylogenetic, population and other kinds of investigations. In our work, we showed that the extinct Syr Darya sturgeon species – *P*. *fedtschenkoi* – was quite distant genetically from the living species *P*. *hermanni* and *P*. *kaufmanni*, and its extinction was a great loss for the biological diversity of the fish fauna of the Ponto-Caspian region.

## Material and Methods

### Samples

DNA samples from the Amu Darya shovelnose sturgeon *P*. *kaufmanni* (KAU02 and KAU03) and dwarf Amu Darya shovelnose sturgeon *P*. *hermanni* (HER01) were obtained from specimens that are stored in 96% ethanol in the Russian reference depository of sturgeon genetic samples (Russian Federal Research Institute of Fisheries and Oceanography, Moscow, Russia).

**Figure 5:**
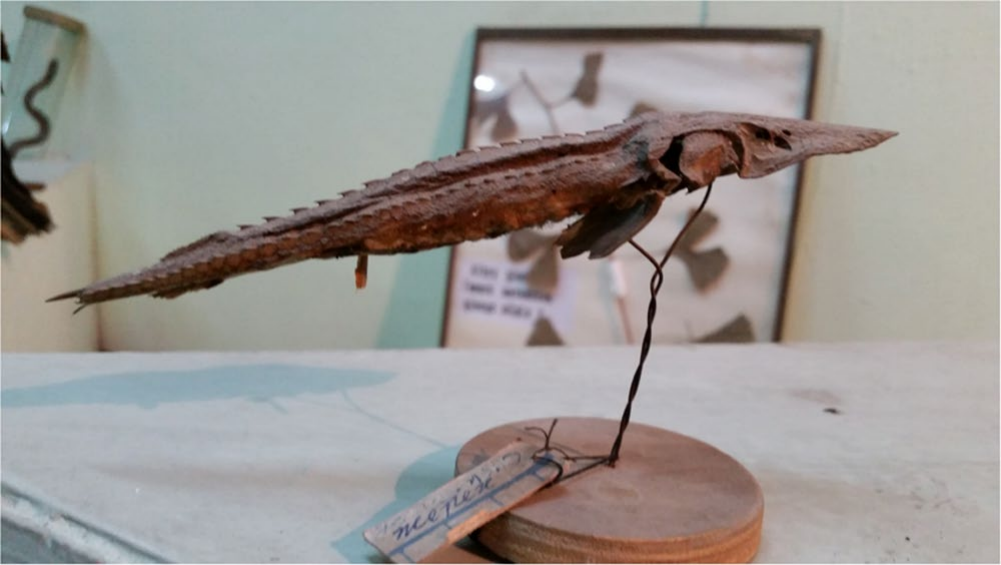
*P*. *fedtschenkoi* specimen displayed in the Museum of Natural History, in Andizhan (Fergana valley, Uzbekistan).

A specimen of the Syr Darya shovelnose was obtained as a dried museum specimen, found in peculiar circumstances. During two years, a joint expedition of the Kazakh Fishery Institute (KazNIIRKh, Almaty) and the Russian Institute of Fishery and Oceanography (VNIRO, Moscow) conducted a field study throughout the Syr Darya river and its tributaries in order to assess the current status of *P*. *fedtschenkoi*. Fish protection authorities, fishermen and merchants at local fish markets were all surveyed about their encounters with this species. In 2015, the Syr Darya basin was explored from the Aral Sea to the Chardara Dam on the border between Kazakhstan and Uzbekistan, and in 2016 the exploration was continued to the Uzbekistan and Kyrgyzstan parts of the Syr Darya basin. There was no live sturgeon fish found, and no recent sightings have been recorded by local fishermen. This data leads us to conclude that this species can be assumed as extinct. During the field expedition, we found a damaged museum specimen at the Museum of Natural history in Andizhan (Fergana valley, Uzbekistan), which was labeled as asp, *Aspius aspius* (Linnaeus, 1758). The museum specimen register book indicates that this item has been on display since the 1960-s. Although this specimen was labeled as and *A*. *aspius*, it resembled a shovelnose, so we received permission to photograph it (Fig. 5) and to collect a small, inconspicuous fragment of fin tissue for genetic verification.

Further evaluation of these photographs revealed that this specimen is indeed a *P*. *fedtschenkoi* (number of dorsal, lateral and abdominal scutes corresponds with the description given by Berg and clearly separates it from two other species of *Pseudoscaphirhynchus* genus (Table 2). This specimen was assigned as FED01.

In total, four samples of *Pseudoscaphirhynchus* were used for whole mitogenome sequencing in this study, which were used to ascertain the correct phylogenetic placement of the Central Asian freshwater endemic sturgeons.

### DNA extraction and sequencing

For Sanger sequencing, DNA from *P*. *kaufmanii* (KAU02) and *P*. *hermanii* (HER01) was extracted using standard phenol-chloroform technique, and complete mtDNA was sequenced using Sanger sequencing. Thirty pairs of primers were designed based on previously published sturgeon genomes to amplify a complete genome by overlapping regions. We performed Sanger sequencing with BigDye v3.0 chemistry from both directions by using the same primers. Primers and PCR conditions were published previously^11^. However, due to the highly degraded DNA quality of *P*. *fedtschenkoi*, PCR amplification of mtDNA with this set of primers mostly failed. To obtain a complete mitochondrial genome of *P*. *fedtschenkoi* sample, we attempted to use Illumina sequencing technology which requires much shorter DNA fragments. For consistency, Illumina sequencing was also performed for *P*. *kaufmanii* and *P*. *hermanii* samples.

For Illumina sequencing, DNA from *P*. *fedtschenkoi* (FED01) was extracted from fin tissue of the museum specimen in the Ancient DNA Facilities of the National Research Center “Kurchatov Institute” (Moscow, Russia), following the previously described methodology^20^. Contemporary DNA samples *P*. *kaufmanii* (KAU03) and *P*. *hermanii* (HER01) were isolated from fin clips preserved in ethanol using a standard phenol-chloroform method of DNA extraction from animal tissue.

Multiplexed DNA-libraries were prepared using an Ovation® Ultralow Library System V2 (NuGEN, USA). Amplified DNA libraries were quantified using a high-sensitivity chip on a 2100 Bioanalyser instrument (Agilent Technologies, USA). The S2 flow cell of Illumina Novaseq6000 genome analyzer (Illumina, USA) was used for DNA-libraries sequencing with 150 bp paired-end reads.

The quality of the NGS data was assessed using FastQC. Data trimming was performed with default parameters using the AdapterRemoval2 tool (version 2.2.2)^21^. K-mer size and Depth-cutoff were set automatically by Norgal^22^. E-value cut-off for BLAST-search was 1e-5. Illumina paired-end reads from FED01, KAU03 and HER01 samples were used for building mtDNA sequence *de-novo* by a Norgal software package^22^.

The resulting mtDNA consensus sequences were annotated using the MitoAnnotator 3.25^23^. The obtained annotation was then used to define partitions in the subsequent phylogenetic analysis.

### Phylogenetic analysis

The phylogenetic analysis for *cytB* sequences was performed for the *P*. *hermanni*, *P*. *kaufmanni* and *P*. *fedtschenkoi* species using our and previously published data^10^. The sequence of *cytB* from starry sturgeon (*A*. *stellatus*, KC130105) was used as an outgroup.

The phylogenetic analyses of the whole mitogenome sequences (excluding control region and *NADH4L* gene) were performed for the *P*. *hermanni*, *P*. *kaufmanni*, and *P*. *fedtschenkoi* species. As an outgroup, we used complete mitochondrial genomes of starry sturgeon – *A*. *stellatus* (NC_005795.1)^16,24^, sterlet sturgeon – *A*. *ruthenus* (KF153104.1), ship sturgeon – *A*. *nudiventris* (KU321568.1), beluga sturgeon – *Huso huso* (NC_005252.1), and mtDNA genomes of other Ponto Caspian sturgeon species, that had been previously been generated^25^.

The phylogenetic relationships for *cytB* gene sequences were reconstructed using the maximum likelihood (ML) method in the MEGA X^26^. The phylogenetic analysis for PCGs (excluding *NADH4L* gene and stop codons) was conducted in IQ-TREE software^27^. The consensus sequence from a multiple alignment and its annotation were generated using NCBI BLAST. Then, a partition file with the coordinates of the PCGs was used in IQ-TREE software (ML method with auto substitution model; number of bootstrap alignments – 1000).

The haplotype network of the *cytB* gene was built by R package Pegas. The alignment of the *cytB* gene of eleven *Pseudoscaphirhynchus* specimens and one *A*. *stellatus* specimen (1140 nucleotides sequences, 51 variable, and 10 informative positions) was loaded in ape R package. Genetic distances of the sequences were estimated by the dist.dna function using Kimura 80 (K80) evolutionary model. The haplotypes were extracted from DNA-alignment using the haplotype function of the Pegas package, and plotted with the haploNet() Pegas function^28^. The genetic distances were estimated by the *dist*.*dna* function of the ape R package^29^. The evolutionary history was inferred by using the Maximum Likelihood (ML) method based on the Hasegawa-Kishino-Yano model with gamma distribution HKY + G model^30^, as was estimated by the “model selection” algorithm of the MEGA X software^26^.

## Supplementary information

Supplementary File.

## Data Availability

All mitogenome assemblies are publicly available at the NCBI BioProject: PRJNA472690.
