# Supplementary material for: Molecular phylogeny of one extinct and two critically endangered Central Asian sturgeon species (genus Pseudoscaphirhynchus) based on their mitochondrial genomes
Source: Sci Rep. 2020 Jan 20;10:722. doi: 10.1038/s41598-020-57581-y (PMC6971001; doi:10.1038/s41598-020-57581-y)
Supplement: Supplementary file 1 — Supplementary File. [file 41598_2020_57581_MOESM1_ESM.docx]

**Supplementary Information**

**Molecular phylogeny of one extinct and two critically endangered Central Asian sturgeon species (genus *Pseudoscaphirhynchus*) based on their mitochondrial genomes.**

Artem V. Nedoluzhko^1*^, Fedor S. Sharko^2,3^, Svetlana V. Tsygankova^2^, Eugenia S. Boulygina^2^, Anna E. Barmintseva^4^, Anna A. Krasivskaya^2^, Amina S. Ibragimova^2^, Natalia M. Gruzdeva^2^, Sergey M. Rastorguev^2**^, Nikolai S. Mugue^4,5**^

^1^Nord University, Faculty of Biosciences and Aquaculture, Bodø, 8049, Norway

^2^National Research Center “Kurchatov Institute”, Moscow, 123182, Russia

^3^Institute of Bioengineering, Research Center of Biotechnology of the Russian Academy of Sciences, Moscow, 117312, Russia

^4^Russian Federal Research Institute of Fisheries and Oceanography, Moscow, 107140, Russia

^5^Koltzov Institute for Developmental Biology RAS, Moscow, 117808, Russia.

* - Corresponding author

** - Equal contribution

Full correspondence address: Dr. Artem V. Nedoluzhko, Nord University, Faculty of Biosciences and Aquaculture, Universitetsalléen 11, 8049, Bodø, Norway.

Phone: +4746901369

e-mail: nedoluzhko@gmail.com

**Supplementary Figures**

**Figure S1.** The read length distribution for the *P. fedtschenkoi* museum specimen (FED01)





**Figure S2.** The read length distribution for the *P. hermanii* (HER01) sample





**Figure S3.** The read length distribution for the *P. kaufmanii* (KAU03) sample.
